# Cannabis and Breastfeeding

**DOI:** 10.1155/2009/596149

**Published:** 2009-04-29

**Authors:** Aurélia Garry, Virginie Rigourd, Ammar Amirouche, Valérie Fauroux, Sylvie Aubry, Raphaël Serreau

**Affiliations:** ^1^Départment d'Ingénierie Biologique, École Polytechnique de l'Université de Nice - Sophia Antipolis, 1645 Route des Lucioles, 06410 Biot, France; ^2^Lactarium d'Ile de France, Institut de Puériculture et de Périnatalogie, 26 Boulevard Brune, 75014 Paris, France; ^3^Centre de Recherche Clinique Paris Centre, 89 rue d'Assas, 75006 Paris, France; ^4^Centre de Recherche Clinique Paris Centre EA 3620, 89 rue d'Assas 75006 Paris, France

## Abstract

Cannabis is a drug derived from hemp plant, *Cannabis sativa*, used both as a recreational drug or as medicine. It is a widespread illegal substance, generally smoked for its hallucinogenic properties. Little is known about the adverse effects of postnatal cannabis exposure throw breastfeeding because of a lack of studies in lactating women. The active substance of cannabis is the delta 9 TetraHydroCannabinol (THC). Some studies conclude that it could decrease motor development of the child at one year of age. Therefore, cannabis use and abuse of other drugs like alcohol, tobacco, or cocaine must be contraindicated during breastfeeding. Mothers who use cannabis must stop breastfeeding, or ask for medical assistance to stop cannabis use in order to provide her baby with all the benefits of human milk.

## 1. Introduction

Breastfeeding is the natural way to feed an infant. It provides benefits to mother and infant and should be the optimal feeding choice for most infants. It contains appropriate amounts of carbohydrates, proteins, fats, minerals, and vitamins, and provides hormones and growth factor as well as maternal antibodies that allow the baby appropriate growth and anti-infection defence. Moreover, breastfeeding helps bonding between mother and child, which is especially important when the mother is a drug addict. In most cases, when the mother uses drugs, the risks incurred by the infant outweigh the benefits of breastfeeding [1]. Therefore, drug abuse poses a threat for the health of both mother and child. 

Nevertheless, cannabis can also be used in therapy. Indeed, medical cannabis can be prescribed for postoperative pain. So far, this medical use of cannabis is legal only in some countries including Canada, Belgium, Australia, the Netherlands, the United Kingdom, New Zealand, Spain and some states of North America. This usage generally requires a prescription. Clinical trials on the therapeutic effects of the cannabis are in progress. A California health care collective has developed a variety of alternative methods of administering medical cannabis orally and externally to avoid smoking which is the most widely used method of administering cannabis. They provide an account of the production, distribution, and administration of nonsmokable cannabis products [2].

The aim of this article is to attempt to answer the question: could cannabis be used during breastfeeding?

## 2. Definition and Epidemiology of Cannabis Use

The species *Cannabis sativa* comes from the hemp plant and is used for various purposes such as fiber, as a drug, as medicine, for oil, as food, and so forth. Cannabis, also called marijuana or haschich, exists in different forms. From a cannabis seedling, three products can be obtained. Marijuana comes from leaves, stems, and flower buds which are dried. It is generally smoked mixed with tobacco, rolled as a cigarette. Haschich is resin obtained from flower buds of the plant presented in compressed plates. It is generally smoked with tobacco and is often cut with other substances more or less toxic. Haschich and marijuana oils are obtained by percolation from haschich purified with an organic solvent or alcohol. Oils contain more cannabinoids than the other forms (30% to 60% of delta 9 TetraHydroCannabinol or THC). They are placed on cigarette paper or added to joints in order to increase their hallucinogenic power.

To determine the current patterns of substance use among youths entering adulthood, sex and age-specific rates of substance use (tobacco, alcohol, cannabis, etc.) in a large sample of French youths aged 12–26 years have been studied. Tobacco, cannabis, and polysubstance use were most frequent among 19–21 year-olds with 10.9% of women who regularly use cannabis, and 4,6% of women who use tobacco with alcohol and cannabis. The age of initiation of tobacco and cannabis use decreased. These results show high prevalence rates of tobacco, alcohol, and drug use in adolescents and young adults in France [3]. There were 1.2 millions regular cannabis users in France in 2005, and half of these were daily users. Regular use has increased against occasional use. According to the drugs addictions and drugs European observatory, France is the leading European country in terms of cannabis use in both adults and youths. Would French breastfeeding women be more concerned about cannabis use during breastfeeding than other European breastfeeding women? [4]. Cannabis is the illicit drug most often used by women of childbearing age. Studies conducted by North American and English teams from 1980 to 2000 show an incidence of cannabis use during pregnancy of 3% to 30% (more often 10% to 15%). The prevalence of substance use among adolescent mothers is significant. To capitalize on the large decrease in use during pregnancy, drug prevention programs for adolescent mothers should target the first 6 months postpartum [5]. In Canada, Fried revealed that 3% of pregnant women regularly use cannabis during pregnancy [6]. 

## 3. Pharmacology Data and Side Effects of Cannabis

Cannabis is a recreational drug usually smoked with tobacco in cigarettes, either on an occasional or regular basis. It contains many compounds (more than 450). The active alkaloid is the delta 9 TetraHydroCannabinol (THC), and its concentration is variable according to the preparations (<0.2% to >20%). Its half-life is 1 to 2.3 days, and traces can persist for up to 4 to 6 weeks. It is rapidly distributed to the brain and adipose tissue. It is stored in fat tissues for long periods (weeks to months) [7].

THC is an agonist of the cannabinoid receptors CB1 located in the nervous system, and CB2 in the brain and some parts of the immune system, notably in the spleen. The endocannabinoid system plays several key roles in pre- and postnatal development. Indeed, during foetal life, the cannabinoid CB1 receptor acts on brain development, regulating neural progenitor differentiation into neurones and glia (as CB2 receptor) and guiding axonal migration and synaptogenesis. Postnatally, CB1 receptor blockade induces oral motor weakness, which reveals a critical role for CB1 receptors in the initiation of milk suckling by neonates [8]. Other observations support the existence of an unknown cannabinoid receptor, with partial control over milk ingestion in newborns [9]. Moreover, the endocannabinoid system interacts with a number of better known molecules involved in appetite and weight regulation, including leptin, ghrelin, and the melanocortins, which leads to the regulation of energy balance and food intake [10]. Cannabinoids are also involved in the expression of key genes for neural development [11]. 

The THC acts on the mesocorticolimbic system via CB1 receptors, which inhibit GABA release, and thus dopaminergic neurons are subsequently not inhibited anymore. The effects of this mechanism are euphoria, modification of lucidity and perceptions, and depersonalization. In animals, THC induces well-defined behavioural effects. Indeed, it produces a characteristic combination of four symptoms: hypothermia, analgesia, hypoactivity, and catalepsy [12]. Other experiments showed distinct effects of cannabis on mice litters, such as lower body weight, when the parents were drugged postnatally during the period of lactation only [13]. In striatum, CB1 receptors decrease dopamine and glutamate release and increase GABA activity in the synapse, which causes locomotor activity slowdown. CB2 receptors are responsible for anti-inflammatory effects, and other therapeutic effects like analgesia, antiemetic effects and decrease of intraocular pressure.

Cannabis causes sedation, weakness, and poor feeding patterns in adult [7]. Moreover cannabis abuse seems to be a risk factor for depressive symptoms [14]. Indeed, frequent cannabis use in teenage girls predicts later depression and anxiety [15]. Cannabis use is also associated with an increased risk of developing schizophrenia [16]. The relative risk for schizophrenia among high consumers of cannabis was 6.0 compared with nonusers [17].

## 4. Cannabis and Breastfeeding

### 4.1. Pharmacokinetic Data

*Cannabis sativa* grows abundantly among other natural vegetation in Pakistan, and it has been reported that buffaloes which graze upon this vegetation produce milk contaminated with THC. Low levels of the major metabolite for THC were found in the urine of children of the region, who were being raised on the milk from these animals. These observations suggest passive consumption of marijuana through milk [18]. 

THC is excreted into human breast milk in moderate amounts [19]. In one feeding, the infant would ingest 0.8% of the weight-adjusted maternal intake of one joint [20]. The ratio milk-to-plasma rates 8 in heavy users [19].

Cannabis may affect the quality and quantity of breast milk [21]. Indeed, studies in animals suggest that marijuana could inhibit lactation by inhibiting prolactin production and, possibly, by a direct action on the mammary glands [7]. Hale reports a positive evidence of risk on milk production. Nevertheless, there are no human data to corroborate these observations. 

### 4.2. Side Effects on Baby and Infant

There are a few studies about the effects of cannabis consumption during lactation on infant health and development. More attention has been directed towards adverse effects of prenatal cannabis exposure.

It is important to note that newborns have also probably been exposed to cannabis during pregnancy. Therefore the effects observed in infant during breastfeeding will be the combined result of foetal and neonatal exposure. Moreover, cannabis is often combined with tobacco in a joint. Thus, side effects of tobacco on infant must be considered. 

THC can accumulate in human breast milk to high concentrations [19], and infants exposed to marijuana through their mother's milk will excrete THC in their urine during 2 to 3 weeks [21]. According to Hale, marijuana could produce sedation and growth delay in infant [7], and a study by Liston have demonstrated that infants exposed to marijuana via breast milk show signs of sedation, reduced muscular tonus, and poor sucking [21].

Moreover, because the important phase of brain growth occurs during baby's first months of life, THC could theoretically alter brain cells metabolism. Animal studies have demonstrated alteration of DNA and RNA synthesis, and proteins needed for proper growth and development impaired in brain cells of newborn animals [21]. 

Two studies evaluated the effects of cannabis use by lactating mother on their child's development. In the first study, no significant differences were found in terms of weaning, growth, and mental or motor development with regard to age [22]. The second study found that cannabis exposure via the mother's milk during the first month postpartum appeared to be associated with a decrease in infant motor development at one year of age. Infants exposed to cannabis for more than half of the days during the first trimester of gestation or the first month of lactation had significantly lower mean Psychomotor Development Index scores than infants with no cannabis exposure (cf.  Table 1). The study did not find a correlation between cannabis exposure during the third month after birth and the motor development. There was no relation between exposure cannabis during the first and the third month after birth and the mental development of the infant. However, it remains a preliminary study, and the cause-effect relation between cannabis exposure during breastfeeding and decrease of the motor development of the infant is not necessarily simple and direct. Other factors come into play like cannabis exposure during pregnancy, passive exposure to cannabis smoke in ambient air, or the quality of the mother-child relationship [23]. There are no studies relating to the long-term effects of marijuana exposure through breast milk. 

Many of these findings remain suggestive due to the number of factors associated with cannabis use which are difficult to control in epidemiologic and clinical studies.

Concerning side effects of tobacco on infants, a study investigated the effects of smoke exposure via mothers' milk and/or via passive smoking during the first year of life. At birth, mean body weight of neonates from smoking mothers was significantly lower than the weight of neonates from nonsmoking mothers. But this difference was no longer significant in infants at 12 months of age. In this study, neither psychomotor nor mental development was affected by smoke exposure during pregnancy and early infancy. Infections of the lower respiratory tract were more frequent in the children of smoking mothers. Breast-fed infants showed concentrations of cotinine 10-fold higher than those who were bottle fed. Nicotine exposure via passive smoking only leads to cotinine excretion in urine of infants higher than adult passive smokers [24]. 

### 4.3. Advice to Drug Addicted Mothers

Cannabis consumption during breastfeeding is contraindicated according to Hale and the American Academy of Pediatrics in Breastfeeding Mothers. If the mother regularly uses cannabis, breastfeeding is contraindicated. If the mother is an occasional user and is not able to stop using cannabis, the situation should be evaluated on a case-by-case basis (age of the baby, quantities absorbed, etc.). Side effects that cannabis can cause must be explained to the mother. Pregnancy and Addictions Studies Group recommends intensive prenatal care, with substitution maintenance programs, by a medico-psychosocial team working in concert with ambulatory health and social workers, to prevent perinatal complications and mother-infant separation.

Moreover, the mother has to avoid cannabis smoke exposure to her child and not to breastfeed during the hours after cannabis use [21]. In all cases, the infant must be carefully monitored, and both mother and baby should follow a psychological and medical survey. 

Mothers should be informed about the risk of sudden infant death syndrome (SIDS) which is associated with maternal smoking during and after pregnancy. The odds ratio for SIDS among normal birth weight infants was approximately 2 for passive exposure (only during infancy), and raised to 3 for combined exposure (during pregnancy and infancy). These data suggest that both intrauterine and passive tobacco exposure are associated with an increased risk of SIDS and are further inducement to encourage smoking cessation among pregnant women and families with children [25]. However, another study has been performed to determine whether maternal or paternal use of drugs during conception, pregnancy, and postnatally increases the risk of SIDS during the first year of the infant's life. Their results show that there was no association between maternal recreational drug use and SIDS. But paternal marijuana use during the periods of conception, pregnancy, and postnatally was significantly associated with SIDS [26].

## 5. Polyintoxications

From all of the above information, cannabis abuse during breastfeeding is not allowed. Mothers who use cannabis may also use other drugs like amphetamine, cocaine, heroine , and so forth. According to the Committee on Drugs of the American Academy of Pediatrics, these drugs belong to drugs of abuse for which adverse effects on the infant during breastfeeding have been reported. For example, cocaine intoxication can cause irritability, vomiting, diarrhoea, tremulousness, and seizures [27]. Therefore, other drug consumption must be taken into account.

## 6. Conclusion

In conclusion, clinical and pharmacokinetic data indicate that cannabis use is dangerous during breastfeeding for the child. Observed effects in breastfed infant like sedation or reduced muscular tonus could be due to, not only cannabis, but also other drugs or medicines (psychotropic, antiepileptic, etc.) that mothers are likely to take. 

Mothers who are drug addicts and want to breastfeed their infant must be sustained by a multidisciplinary team. A substitution treatment like methadone should be chosen in order to stop drug use and to protect their child from drug side effects. Medical teams must be advised about medication, alcohol, and other drugs abuse that could be contraindicated with breastfeeding. The Medic-Al network provide health professionals and patients with up-to-date information [28]. Our references about drugs and breastfeeding are (Addictions and Pregnancy Studies Group (GEGA), Regional Pharmacovigilance Centre (CRPV), Reference Centre on Teratogenic Agents (CRAT), and Mother Risk Program, etc.). Medic-Al network will facilitate discussion about pharmacokinetic data in maternal milk and allow comparative study about potential benefits and risks. 

## Figures and Tables

**Table 1:** Mean motor development scores* corresponding with cannabis exposure during gestation and lactation [23].

	Motor Development PDI
Days of infant Cannabis exposure	Mean

*Trimester one*	
0	**105** (*n* = 84)
1 to 44	99 (*n* = 36)
45 to 90	**93** (*n* = 16)

*Trimester two*	
0	102 (*n* = 98)
1 to 44	104 (*n* = 25)
45 to 90	94 (*n* = 13)

*Trimester three*	
0	101 (*n* = 94)
1 to 44	107 (*n* = 28)
45 to 90	94 (*n* = 14)

*Lactation month one*	
0	**102** (*n* = 81)
1 to 44	106 (*n* = 38)
45 to 90	**90** (*n* = 17)

*Lactation month three*	
0	102 (*n* = 84)
1 to 44	103 (*n* = 28)
45 to 90	97 (*n* = 13)

*Motor developmental scores (Psychomotor Development Index, PDI) were from Bayley Scales of Infant Development. During the first trimester, PDI decreased with increasing cannabis exposure (test for linear trend, *P* = .005). During the first lactation month, mean PDI for the higher exposure group was lower than the PDI for the no or low exposure groups (one-way ANOVA *P* = .008).
